# A Streamlined Protocol for Developing a Clinicopathological Prediction Model for Patient Survival of Post‐Resection of Pancreatic Cancer

**DOI:** 10.1002/cam4.71535

**Published:** 2026-01-28

**Authors:** Yi Ma, Eunice Lee, Khashayar Asadi, Mehrdad Nikfarjam, Hong He

**Affiliations:** ^1^ Department of Surgery University of Melbourne, Austin Precinct Melbourne Victoria Australia; ^2^ Department of General Surgery Monash Health Melbourne Victoria Australia; ^3^ Department of Hepato‐Pancreato‐Biliary Surgery Austin Health Melbourne Victoria Australia; ^4^ Department of Anatomical Pathology Austin Health Melbourne Victoria Australia

**Keywords:** CD4+, CD8+, CK19, MHC I, overall survival, pancreatic cancer, post‐resection

## Abstract

**Background:**

Pancreatic ductal adenocarcinoma (PDA) is one of the most malignant solid cancers. As surgery is the only cure, prediction of long‐term survival post‐resection is critical to guide patient selection for the subsequent treatment. Tumour immune evasion plays a key role in PDA tumorigenesis.

**Materials and Methods:**

Using a streamlined protocol, we developed a clinicopathological prediction model for the overall survival of patients with PDA after resection by integrating tumour immunological features and clinical data. Multiplex immunohistochemistry was performed using human tumour microarray samples. The results were combined with retrospectively collected clinical data of 79 patients. Variables were selected by least absolute shrinkage and selection operator (LASSO) regression with 10‐fold cross‐validation to develop the prediction model. The performance of the model was assessed using the concordance index, receiver operating characteristic curve, calibration plot and decision curve analysis. The model was validated by bootstrap resampling.

**Results:**

Cancer cell MHC I intensity, CD4+ T cell to tumour cell ratio, resection margin status and tumour T stage were identified for prediction model development using Cox proportional hazard regression. Discrimination of developed model was moderate on the time‐dependent area under curve at one (0.698), three (0.765) and five (0.825) years. A small decrease in the overall c‐index from 0.67 to 0.652 was shown in the internal validation by bootstrapping.

**Conclusion:**

Our protocol provided a framework for developing a complex model that will significantly contribute to clinical practice.

## Graphical abstract

We have identified the spatial distribution of MHC‐I, CD4, CD8, CD19, PAK4, and LC3B using multiplex immunostaining. We then combined the data of multiplex immunostaining with clinical pathological data for the software analysis to create a model that can be used to predict the post‐resection survival of pancreatic cancer patients.

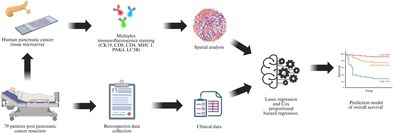

**Abbreviations**
AIC: Akaike information criterion
AJCC: American joint committee on cancer
AUC: area under curve
CD: cluster of differentiation
CK: Cytokeratin
Cox PH regression: Cox proportional hazard regression
CV: cross validation
DCA: decision curve analysis
Dxy: Somer's delta
IQR: inter‐quantile range
LC3B: microtubule‐associated protein 1 light chain 3 beta
MHC: major histocompatibility complex
mIHC: multiplex immunohistochemistry
PAK: P21‐activated kinase
PCA: principal component analysis
PDA: pancreatic ductal adenocarcinoma
ROC curve: receiver operating characteristic curve
RT: room temperature
TMA: tissue microarray
TME: tumour microenvironment
TRIPOD: transparent reporting of a multivariable prediction model for individual prognosis or diagnosis
VIF: variance inflation factor

## 1 Introduction

Pancreatic ductal adenocarcinoma (PDA) is one of the most malignant solid cancers, with less than 10% five‐year survival [1]. While surgery remains the only hope of cure, 50%–60% of patients have metastatic disease upon diagnosis, thus unsuitable for surgery [2]. Even with successful surgical resection, most patients develop cancer recurrence, and only 25% of patients can survive more than 5 years [2]. Given the poor prognosis, the prediction of post‐resection survival in individual patients will be important for patient selection for adjuvant therapies.

The role of tumour microenvironment (TME) in PDA tumorigenesis is gaining increasing attention in targeted treatment of PDA, and one of the major aspects of TME is immune cell infiltration and function [3]. However, PDA down‐regulates anti‐tumour immunity by multiple mechanisms, such as increased infiltration of immunosuppressive cells and decreased infiltration of helper and cytotoxic T cells [4]. Furthermore, PDA cells can degrade cell surface MHC I molecules through autophagy, which reduces antigen presentation for CD8+ T cell activation [5]. Increasing evidence has also pointed to an important role of p21‐activated kinase (PAK), a family of serine/threonine kinases that function downstream of RAS protein, in cancer immune evasion [4]. Among the PAK family members, PAK4 was shown to inhibit T cell infiltration and down‐regulates cancer cell surface MHC I expression [6, 7]. Therefore, PAK4 expression, cancer cell autophagy level and cell surface MHC I expression are likely important biomarkers for T cell infiltration and function in PDA. Furthermore, multiple studies have confirmed the prognostic value of tumour infiltrating lymphocytes in oncological outcomes of PDA such as patient survival [8, 9].

Given the important role of immune cells in PDA, the development of predictive models integrating both clinical data and tumour immunological features has been attempted using different techniques [10, 11, 12]. A 2018 study utilised sequential immunohistochemistry staining for tumour infiltrating T cells, macrophages and tumour stromal markers in human PDA specimens to build a prognostic signature of progression free survival of PDA patients, but did not integrate clinical considerations in the prognostic model [11]. On the other hand, a later study thoroughly analysed the TME of human PDA specimens using CODEX, which is a barcoding multiplex immunofluorescence technique for spatial analysis of tissue specimens, and integrated the information collected with clinical variables of patients by six different machine learning algorithms to compare their predictability of survival [12]. However, the availability and cost of the cutting‐edge technology as well as advanced data science skills may limit its potential for clinical translation. In comparison, Chen et al. [10] used sequential multiplex immunofluorescence staining for spatial analysis of tumour infiltrating CD8+ T cells and macrophages in human PDA samples, and combined this with clinical variables of patients to develop a prediction model based on univariate and multivariable regression models. While it is a much more simplified technique than CODEX and is more ready for clinical translation, multivariable regression analysis is susceptible to covariation between variables which may affect variable selection and prediction model development.

Based on the literature, we believe a more readily available and cost‐effective technique for immune cell spatial profiling should be combined with an easy to implement but stringent method for variable selection to develop a prediction model with clinical translation potential. In this study, we assessed the feasibility of developing and validating a clinicopathological predictive model for the overall survival of post‐resection PDA patients using a standardised but easy to implement protocol in analysing multiplex immunohistochemistry (mIHC) results and selecting independent variables.

## 2 Material and Methods

The Transparent reporting of a multivariable prediction model for individual prognosis or diagnosis (TRIPOD) statement was followed for model development and validation [13].

### 2.1 Patient Information Collection and Tissue Microarray Generation

The collection of patients' clinical information and generation of human PDA tissue microarray (TMA) were approved by the Austin health human research ethics committee (HREC/73948/Austin‐2021) as previously described [6]. Informed consent from study participants was waived due to the retrospective and de‐identified nature of the study. Research involving human participants was conducted in accordance with the declaration of Helsinki.

Adult patients (age over 18 years) who had surgical resection of PDA between 2008 and 2019 at Austin health (an Australian tertiary care centre) were identified. Surgical resections included pancreaticoduodenectomy, distal pancreatectomy or total pancreatectomy by either open or laparoscopic techniques. Patients who had premature mortality from either delayed surgical complications, background comorbidities or incorrect disease staging were excluded. Once recruited, participants' age, sex, cancer site (pancreatic head/neck, body/tail or multifocal), TNM staging, tumour pathological grade, evidence of lymphovascular or perineural invasion, the status of portal vein resection, adjuvant chemotherapy received, survival status and duration (in months) were retrospectively reviewed and collected from medical records. Modified 8th edition American joint committee on cancer (AJCC) staging system for PDA was used for TNM staging [14].

Corresponding formalin‐fixed PDA tumour blocks of patients were assessed by a qualified anatomical pathologist, and three 1 mm diameter cores were taken from each tumour sample to assemble TMA blocks.

### 2.2 Multiplex Immunohistochemistry

The TMA blocks were sectioned into 5 μM slides for multiplex immunohistochemistry (mIHC) staining. mIHC staining was conducted using the Opal 6‐Plex Manual Detection Kit (Akoya Biosciences, USA) following the manufacturer's protocol. After dewaxing and rehydration, samples were boiled in Tris‐EDTA buffer (10 mM Tris base, 1 mM EDTA solution, 0.05% Tween 20, pH 9.0) at 99°C for 30 min and then blocked with 1× Antibody Diluent/Block (Akoya Biosciences, USA) at room temperature (RT) for 10 min. Primary antibodies (Table S1) were added to samples and incubated at RT for an hour before 10 min of incubation with 1× Opal Anti‐Ms + Rb HRP (Akoya Biosciences, USA) at RT. The samples were then stained with Opal fluorophores (Akoya Biosciences, USA) diluted in 1× Plus Manual Amplification Diluent (Akoya Biosciences, USA). This sequence was repeated for all primary antibodies and Opal fluorophore pairs (Table S2) before DAPI counterstaining for 5 min at RT.

**FIGURE 1:**
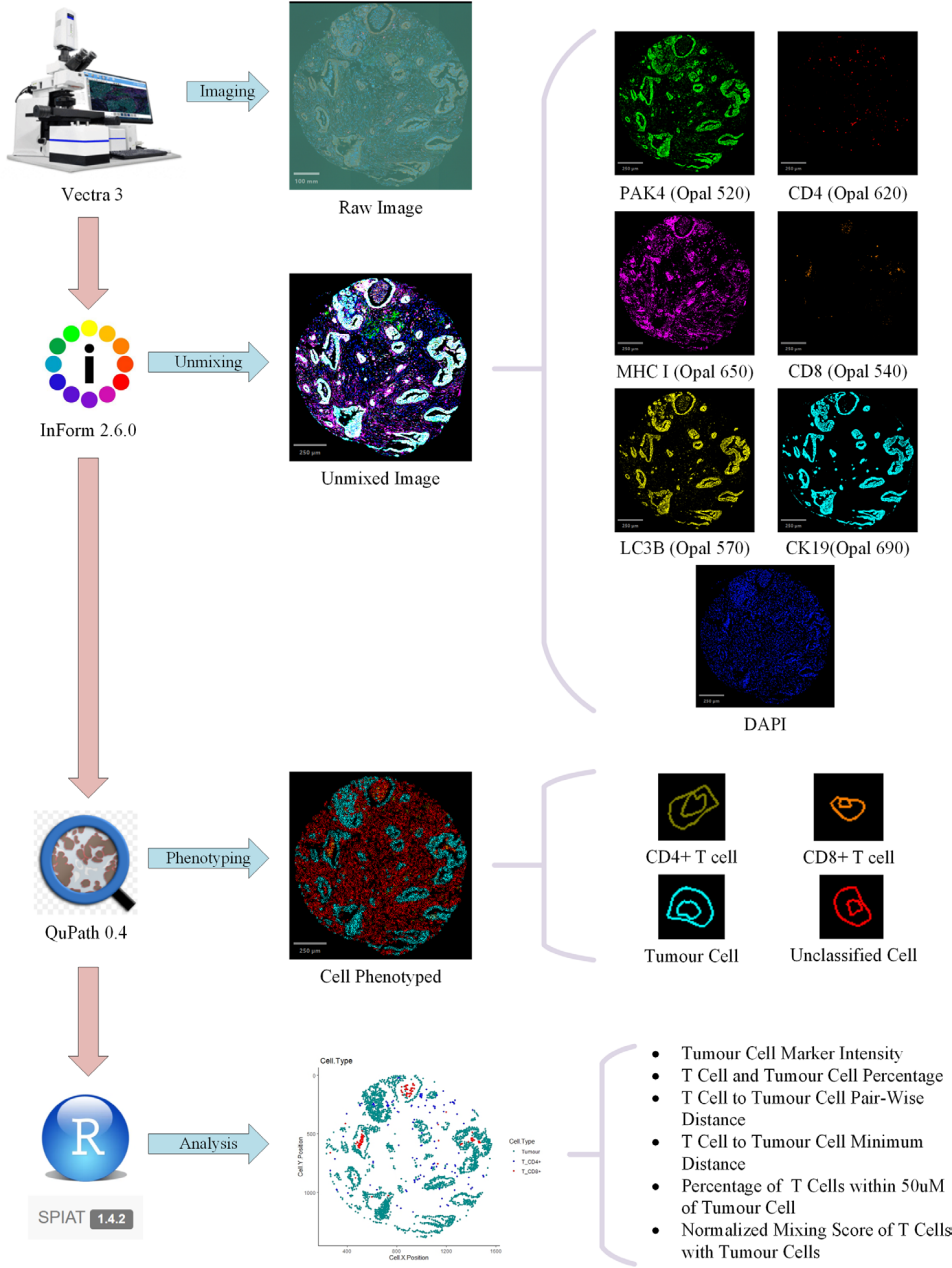
Flow diagram demonstrating analysis steps of multiplex immunohistochemistry of human tissue microarray samples. Raw images were captured by Vectra 3 platform and unmixed by InForm software using predetermined spectral library. CD4+, CD8+, and Tumour cells were then phenotyped by QuPath software based on marker intensity. The cell map was constructed and spatially analysed using SPIAT package in R, with output variables listed.

The process for image capturing and analysis was summarised in Figure 1. The whole slide images were captured using Vectra 3 Automated Quantitative Pathology Imaging System (Akoya Biosciences, USA). Raw images were then unmixed against the pre‐developed Opal fluorophores spectral library into single marker images using InFrom version 2.6.0 (PerkinElmer, USA). Cells were detected by DAPI nuclear staining and classified into CD4+ T cells, CD8+ T cells, and CK19+ Tumour cells in QuPath version 0.4.3 [15]. Spatial analysis was conducted by SPIAT package version 1.4.2 under R version 4.4.1 to determine the following variables: (1) Tumour cell PAK4, LC3B, and MHC I mean intensity; (2) Percentages of CD4+ T cells, CD8+ T cells, and tumour cells within TMA core; (3) Average pairwise distance and minimum distance between CD4+ or CD8+ T cells and tumour cells, respectively; (4) Percentage of CD4+ or CD8+ T cells within 50 μM radius of tumour cells, respectively; (5) Normalised mixing score of CD4+ or CD8+ T cells with Tumour cells [16, 17]. Ratios of CD4+ or CD8+ T cells to tumour cells were calculated based on percentages of cells stained.

### 2.3 Development of a Prediction Model

**FIGURE 2:**
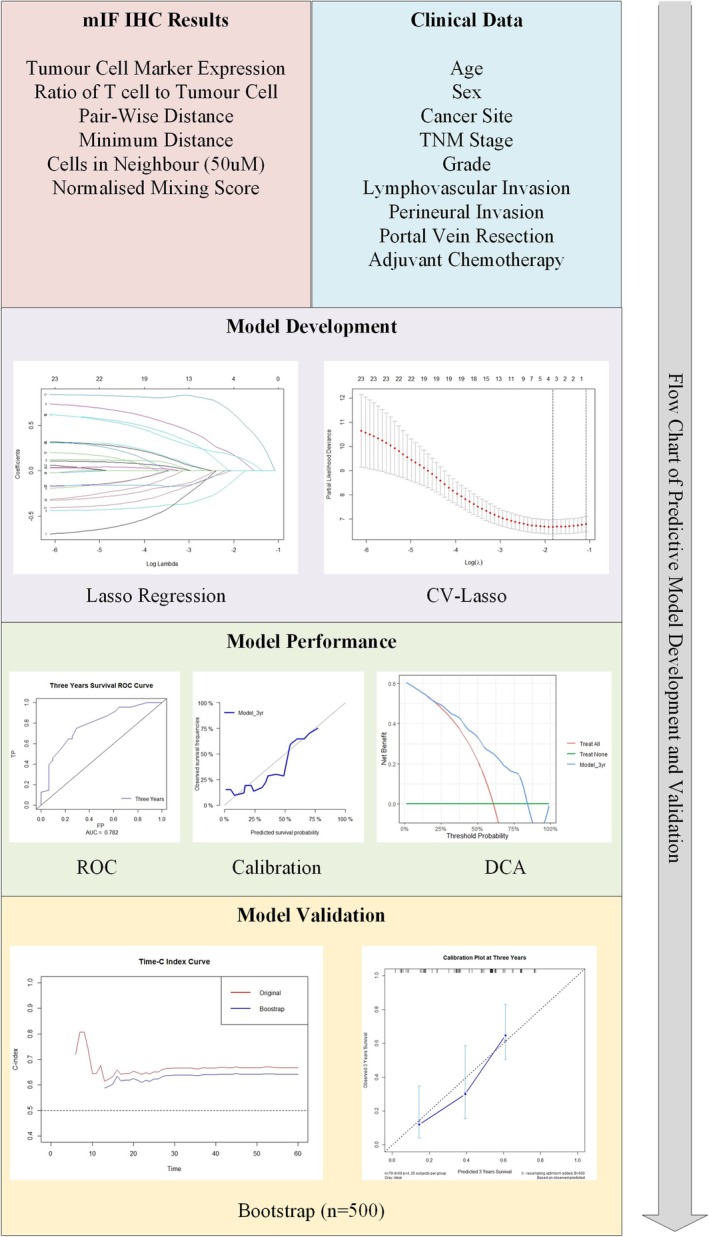
Flow chart of predictive model development and validation. Input variables were obtained from both multiplex immunohistochemistry results and retrospectively collected clinical data. Lasso regression with ten folds cross validation was then applied for variable selection. Model performance was then assessed by Receiver Operating Characteristic (ROC) curve, calibration plot and Decision Curve Analysis (DCA). The model was then validated for both concordance index (c‐index) and calibration using bootstrap resampling (*n* = 500).

The process for developing and validating the clinicopathological prediction model was summarised in Figure 2. The outcome variable for the prediction model was defined as overall patient survival from the date of surgery to December 2023. There were two types of predictor variables: clinical variables from a retrospective review of medical records and mIHC variables from spatial analysis (Figure 2). Patients were divided into under and above 65 for comparison. The mIHC variables were converted into low versus high expressions by median due to several considerations: (1) The semi‐quantitative nature of the technique; (2) Significant multicollinearity between the variables as demonstrated by the variance inflation factor (VIF, Table S3); (3) Consideration of biological meaning and result interpretation. As shown in Figure S1, transformation of the variables by principal component analysis (PCA) was performed, but the biological meaning of the principal components was difficult to interpret. Table S4 showed VIF of the binary variables after transformation, which lowered from above 5 pre‐transformations to below 5 post‐transformations. VIF and PCA were conducted by regclass, stats and psych R packages [16, 18, 19]. The ratio of T cells to tumour cells was used instead of cell percentages to show the differences.

A prediction model was developed using glmnet, survival and rms R packages [16, 20, 21, 22, 23]. A complete case analysis was conducted by eliminating participants with missing data in any variables. least absolute shrinkage and selection operator (LASSO) regression with 10‐fold cross‐validation (CV) was employed for variable selection and correlation coefficient estimation by using log Lambda with minimum partial likelihood deviance [21, 24]. Cox Proportional Hazard (PH) regression model was then constructed using selected variables and their corresponding correlation coefficients [20, 22].

### 2.4 Assessing the Performance of the Prediction Model

As shown in Figure 2, the performance of the prediction model was assessed across discrimination, calibration, potential clinical benefit, and survival analysis using the survivalROC, pec, dcurves, survminer, and survival R packages [16, 22, 25, 26, 27, 28]. Discrimination of the model was measured by overall concordance index (c‐index) and time‐dependent receiver operating characteristic curve (ROC) at 1, 3, and 5 years post‐resection [22, 25]. The area under the curve (AUC) was calculated for all three time points [25]. Calibration was visualised by plotting predicted against observed survival probabilities for 1, 3, and 5 years post‐resection [26]. The potential clinical benefit of the prediction model was assessed by decision curve analysis (DCA), which compares the model with “treat‐all” and “treat‐none” strategies across threshold probabilities for case diagnosis [27, 29]. Finally, participants were classified into low versus high‐risk groups based on risk scores estimated by the prediction model. The difference in survival between the two groups was determined by the Kaplan–Meier curve with a log‐rank test for statistical significance [28].

### 2.5 Validation of the Performance of the Prediction Model

Internal validation of the prediction model was performed with bootstrap resampling (Figure 2) [30]. The prediction model was internally validated for discrimination and calibration by the built‐in bootstrap function of the rms and pec R packages [16, 20, 26]. The overall c‐index of the bootstrapped model, as well as the time‐dependent c‐index up to 5 years post‐resection, was assessed and compared with the original model for validation of model discrimination [20, 26]. A calibration plot of the bootstrapped model was also constructed with a 95% confidence interval demonstrated [20].

### 2.6 Statistical Analysis

Median ± inter‐quantile range (IQR) was reported for continuous variables due to skewed distribution of multiple variables. The number of events and percentage of total events were reported for binary, ordinal and categorical variables. Univariate and multivariable Cox PH regressions were employed to assess correlation with survival, with hazard ratios and two‐sided *p*‐values of variables reported. *p*‐value below 0.05 was considered statistically significant. Statistical analysis was conducted with R version 4.4.1 unless otherwise stated [16].

## 3 Results

### 3.1 Recruitment of Study Participants and Summary of Study Variables

Tumour microarray (TMA) cores of PDA patients were stained for PAK4, LC3B, MHC I, CD4, CD8 and CK19 (Figure 1). The choice of markers was based on the role of CD4+ and CD8+ T cells in anti‐tumour immunity and the interaction between cancer cell surface MHC I and T cell receptor for CD8+ T cell activation [3, 31]. Furthermore, recent evidence also pointed to the role of cancer cell autophagy in the regulation of cell surface MHC I, and thus, LC3B was selected as a marker of autophagy level [5]. PAK4 (p21‐activated kinase 4), on the other hand, was recently found to regulate T‐cell infiltration and function in PDA and thus was included [6].

**FIGURE 3:**
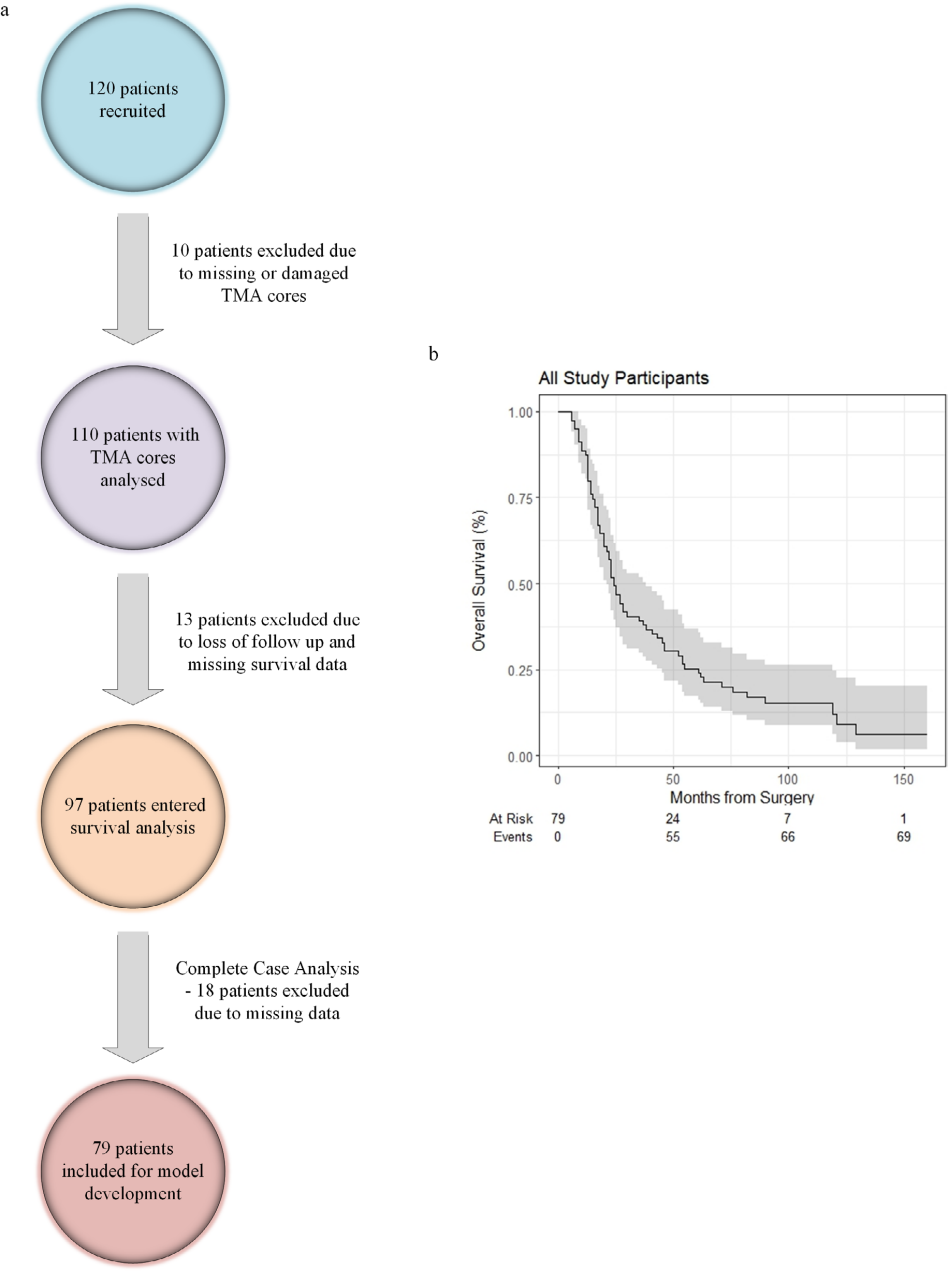
Study participant recruitment process and survival analysis. (a) Flow chart showing the process of study participant recruitment and exclusion. A complete case analysis was conducted for model development. (b) Kaplan–Meier curve analysis of all study participants with numbers at risk demonstrated.

The patient selection was shown in Figure 3a. Medical record search identified 120 patients as per the inclusion criteria, and 41 patients were excluded due to: (1) missing or damaged TMA cores; (2) loss of follow‐up and missing survival data; (3) missing clinical data. The remaining 79 patients with complete sets of variables were recruited for the development of the prediction model. The overall survival of all participants was shown in the Kaplan–Meier Curve (Figure 3b).

The medians and IQR of the mIHC variables were summarised in Table 1. A summary of all variables used for developing the prediction model was shown in Table 2. Among the TNM stages, the M stage was not included as none of the recruited patients had metastatic disease at resection. Furthermore, none of the patients had visible residual tumours on the resection margin (i.e., R2 resection margin) (Table 2).

**TABLE 1:** Cut‐off values of mIF IHC results.

Variable (*n* = 79)	Median	IQR
PAK4 intensity	0.206	0.149
LC3B intensity	0.612	0.665
MHC I intensity	0.878	1.17
% CD4+ T cells	16.6	15.9
% CD8+ T cells	9.91	11.3
% Tumour cells	72.0	27.6
CD4:CK19 ratio	0.271	0.453
CD8:CK19 ratio	0.178	0.240
CD4:CK19 pair‐wise distance	563	74.5
CD8:CK19 pair‐wise distance	571	79.2
CD4:CK19 minimum distance	89.4	54.8
CD8:CK19 minimum distance	103	68.1
% CD4 within 50 μM from CK19	4.83	6.04
% CD8 within 50 μM from CK19	2.38	3.58
CD4:CK19 normalised mixing score	0.203	0.234
CD8:CK19 normalised mixing score	0.217	0.219

**TABLE 2:** Summary of study variables.

Variable (*n* = 79)	Median/number	IQR/percentage
Age		
Below 65	41	51.90
Above 65	38	48.10
Sex		
Male	37	46.84
Female	42	53.16
PAK4 intensity		
Low	40	50.63
High	39	49.37
LC3B intensity		
Low	39	49.37
High	40	50.63
MHC I intensity		
Low	40	50.63
High	39	49.37
% CD4+ T cells		
Low	39	49.37
High	40	50.63
% CD8+ T cells		
Low	40	50.63
High	39	49.37
% Tumour cells		
Low	39	49.37
High	40	50.63
CD4:CK19 ratio		
Low	40	50.63
High	39	49.37
CD8:CK19 ratio		
Low	39	49.37
High	40	50.63
CD4:CK19 pair‐wise distance		
Low	39	49.37
High	40	50.63
CD8:CK19 pair‐wise distance		
Low	39	49.37
High	40	50.63
CD4:CK19 minimum distance		
Low	40	50.63
High	39	49.37
CD8:CK19 minimum distance		
Low	40	50.63
High	39	49.37
%CD4 within 50 μM from CK19		
Low	40	50.63
High	39	49.37
%CD8 within 50 μM from CK19		
Low	39	49.37
High	40	50.63
CD4:CK19 normalised mixing score		
Low	39	49.37
High	40	50.63
CD8:CK19 normalised mixing score		
Low	39	49.37
High	40	50.63
Cancer site		
Head/neck	73	92.41
Body/tail	5	6.33
Multifocal	1	1.27
Resection margin		
R0	52	65.82
R1	27	34.18
R2	0	0
T stage		
T1	12	15.19
T2	52	65.82
T3	14	17.72
T4	1	1.27
N stage		
N0	23	29.11
N1	31	39.24
N2	25	31.65
Grade		
1	2	2.53
2	32	40.51
3	45	56.96
Lymphovascular invasion		
No	22	27.85
Yes	57	72.15
Perineural invasion		
No	52	65.82
Yes	27	34.18
Portal vein resection		
No	58	73.42
Yes	21	26.58
Adjuvant chemotherapy		
No	13	16.46
Yes	66	83.54
Death		
No	10	12.66
Yes	69	87.34
Survival in months	24	42.5

Abbreviation: IQR, interquantile range.

### 3.2 Identification of Variables Using Univariate and Multivariable Cox Proportional Hazard Regression Analysis

Univariate and multivariable Cox PH regression were subsequently conducted for all variables to assess if they are independent prognosticators. In univariate regression, out of the clinical variables, only resection margin status (HR 2.82, *p* < 0.001) and tumour T stage (1.61, *p* < 0.008) were found to be significantly correlated with overall survival (Table 3). For the mIHC variables, CD4+ T cell to CK19+ tumour cell ratio was found to be negatively correlated with overall survival (HR 1.68, *p* < 0.036) while CD8+ T cell to CK19+ tumour cell ratio had an insignificant effect (HR 0.89, *p* = 0.6) (Table 3). The remaining measures of tumour cell markers and T cell and tumour cell interaction did not seem to affect prognosis on univariate analysis (Table 3).

**TABLE 3:** Univariate and multivariable Cox regression results of study variables.

Variable (*n* = 79)	Univariate		Multivariable	
	Hazard ratio	*p*	Hazard ratio	*p*
Age				
Below 65	—	—	—	—
Above 65	1.18	0.5	1.18	0.6
Sex				
Male	—	—	—	—
Female	0.78	0.3	0.70	0.3
PAK4 intensity				
Low	—	—	—	—
High	1.11	0.7	1.27	0.5
LC3B intensity				
Low	—	—	—	—
High	0.74	0.2	1.05	> 0.9
MHC I intensity				
Low	—	—	—	—
High	0.71	0.2	0.84	0.6
CD4:CK19 ratio				
Low	—	—	—	—
High	1.68	0.036	2.54	0.026
CD8:CK19 ratio				
Low	—	—	—	—
High	0.89	0.6	0.25	0.010
CD4:CK19 pair‐wise distance				
Low	—	—	—	—
High	0.91	0.7	1.52	0.3
CD8:CK19 pair‐wise distance				
Low	—	—	—	—
High	1.01	> 0.9	0.62	0.2
CD4:CK19 minimum distance				
Low	—	—	—	—
High	1.25	0.4	0.75	0.5
CD8:CK19 minimum distance				
Low	—	—	—	—
High	1.36	0.2	1.05	> 0.9
%CD4 within 50 μM from CK19				
Low	—	—	—	—
High	1.16	0.5	0.93	0.9
%CD8 within 50 μM from CK19				
Low	—	—	—	—
High	0.87	0.6	1.28	0.6
CD4:CK19 normalised mixing score				
Low	—	—	—	—
High	0.86	0.5	1.75	0.3
CD8:CK19 normalised mixing score				
Low	—	—	—	—
High	0.77	0.3	0.40	0.10
Cancer site				
Head/neck	—	—	—	—
Body/tail	1.05	> 0.9	5.09	0.035
Multifocal	2.16	0.5	0.10	0.092
Resection margin				
R0	—	—	—	—
R1	2.82	< 0.001	2.45	0.017
T stage	1.61	0.008	2.12	0.016
N stage	1.15	0.4	0.92	0.7
Grade	1.21	0.4	1.33	0.4
Lymphovascular invasion				
No	—	—	—	—
Yes	1.51	0.15	1.79	0.12
Perineural invasion				
No	—	—	—	—
Yes	1.42	0.2	1.09	0.8
Portal vein resection				
No	—	—	—	—
Yes	1.47	0.2	1.50	0.3
Adjuvant chemotherapy				
No	—	—	—	—
Yes	0.57	0.080	0.35	0.019

As univariate regression does not control for confounding and suppression effects between variables, multivariable regression was performed. As shown in Table 3, in contrast to univariate regression results, both CD4+ T cell and CD8+ T cell to CK19+ Tumour cell ratios were found to be significantly correlated with overall survival but in different directions (HR 2.54, *p* = 0.026 vs. HR 0.25, *p* = 0.010). This suggested that the ratios between T cells and tumour cells are independent prognosticators of patient overall survival (Table 3). Among the clinical variables, resection margin status (HR 2.45, *p* = 0.017), tumour T stage (HR 2.12, *p* = 0.016), tumour location (HR 5.09, *p* = 0.035) and whether a patient had adjuvant chemotherapy (HR 0.35, *p* = 0.019) are significantly correlated with overall survival (Table 3). R2 resection margin was not included for statistical analysis in Table 3 as none of the study participants have R2 resection margin (Table 2).

As American joint committee on cancer (AJCC) cancer staging system is a well‐established tool to assess cancer prognosis and guide cancer treatment, we also staged the patients based on AJCC eighth edition pancreatic cancer stages and assess its performance in our study population [32]. However, as shown in Figure S2, despite a trend existing between AJCC stages and overall survival by log‐rank test, it failed to reach significance (*p* = 0.1). This is further confirmed in univariate and multivariable Cox PH regression analysis (Table S5). However, this lack of significance can be related to the small sample size and thus lack of power.

### 3.3 Development of a Clinicopathological Prediction Model for Overall Survival

While multivariable Cox PH regression identified the independent prognosticators for overall survival, it is affected by inter‐correlation between predictors and may lead to model overfitting for prediction model development [33]. A more effective and stringent technique for the selection of predictor variables and prediction model development is LASSO regression with L1 regularisation [24].

**FIGURE 4:**
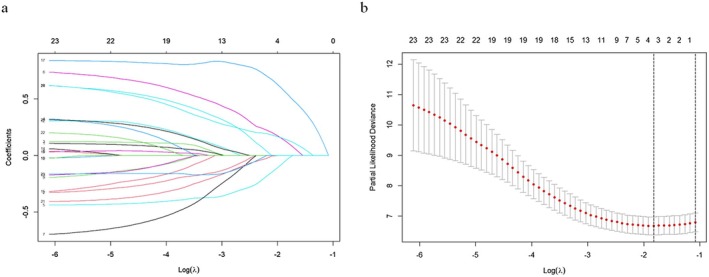
Model development by cross validated lasso regression. (a) Lasso regression curve of all variables showing coefficients versus log Lambda (*λ*). (b) Ten‐fold cross validation (CV) of Lasso regression was conducted, and log Lambda with minimum Partial Likelihood Deviance was used to select variables.

All clinical and mIHC variables were included for LASSO regression, with log lambda plotted against the correlation coefficient, and each line represented a single variable (Figure 4a). Ten‐fold cross‐validation (CV) of LASSO regression was performed to select the optimal number of variables for the prediction model. The log Lambda versus partial likelihood deviance plot for CV LASSO regression and the log Lambda that correlated with minimum partial likelihood deviance were used for variable selection (Figure 4b). Four predictor variables including MHC I Intensity on tumour cells, CD4+ T cell to CK19+ tumour cell ratio, resection margin status, and tumour T stage were identified based on a Lambda of 0.167 (Table 4). Their correlation coefficients were also provided by CV LASSO regression (Table 4).

**TABLE 4:** Variables Identified by CV Lasso Regression.

Variables (*n* = 79)	Coefficients	Hazard ratio
MHC I Intensity		
Low	—	—
High	0.032	0.97
CD4:CK19 Ratio		
Low	—	—
High	0.116	1.12
Resection Margin		
R0	—	—
R1	0.545	1.72
T Stage	0.138	1.15

*Note:* Coefficients for selection: n.fold = 10; Lambda.min = 0.167. Coefficients of model: AIC = 492.188; C‐index = 0.67.

The prediction model for overall survival was then constructed by Cox PH regression using the identified predictor variables. Hazard ratios for each variable were calculated based on their correlation coefficients, with the Akaike information criterion (AIC) of the model estimated to be 492.188 and an overall c‐index of 0.67 (Table 4). Risk score can then be calculated by using the Cox PH regression equation.

### 3.4 Assessment of Discrimination and Calibration of the Prediction Model

**FIGURE 5:**
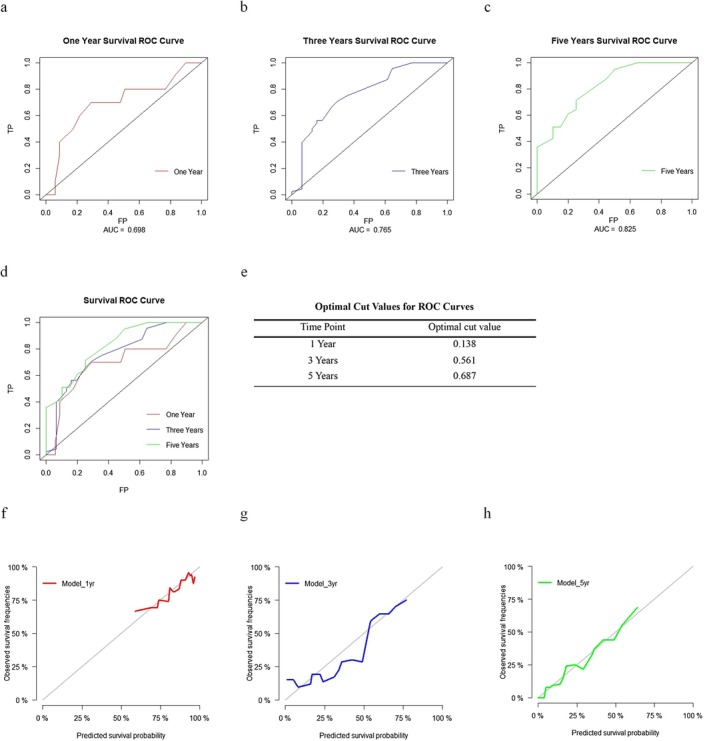
Assessing the performance of the prediction model. (a–c) Time dependent Receiver Operating Characteristic (ROC) curve of predictive model at 1, 3 and 5 years with Area Under Curve (AUC) as calculated. (d) 1, 3 and 5 years ROC curve was combined for direct comparison. (e) Optimal cut‐off values of model risk score at 1, 3 years and give years. (f–h) Calibration plot of prediction model at 1, 3 and 5 years respectively, showing correlation between predicted and observed survival probabilities.

While the c‐index is equivalent to the AUC for assessment of discrimination in logistic regression, this is not the case in Cox PH regression due to time‐predicted risks [34]. As shown in Figure 5a–c, the time dependent ROC curve was constructed at 1, 3 and 5 years, with AUC calculated for all three time points (0.698, 0.765 and 0.825, respectively). The rising AUC from 1 to 5 years suggested an improvement of the model to discriminate between outcomes over time (Figure 5d). The number of survived participants ranged from 70, 31 to 20 over 1, 3 and 5 years respectively due to patient deaths, which suggested sufficient event rates for assessment of model performance within the limitation of the sample size. Furthermore, the optimal cut‐off values of risk scores for the classification of events were shown in Figure 5e.

To determine the correlation between predicted and observed survival probability, calibration plots of the prediction model at 1, 3 and 5 years were constructed and shown in Figure 5f–h respectively. The predicted survival probabilities by the prediction model correlated well with the observed survival probabilities at all three time points, suggesting the model is well‐calibrated (Figure 5f–h).

We also compared the performance of our current model against AJCC stages by assessing the discrimination of AJCC stages on overall survival in the study cohort. As shown in Figure S3, time dependent ROC curves were created for AJCC stages at 1, 3, and 5 years, with AUC reported as 0.516, 0.587, and 0.62 respectively. This suggests the AJCC stages performed poorly as the sole predictor of patient survival in the current study cohort in comparison to the developed prediction model.

### 3.5 Internal Validation of the Prediction Model by Bootstrap Resampling

**FIGURE 6:**
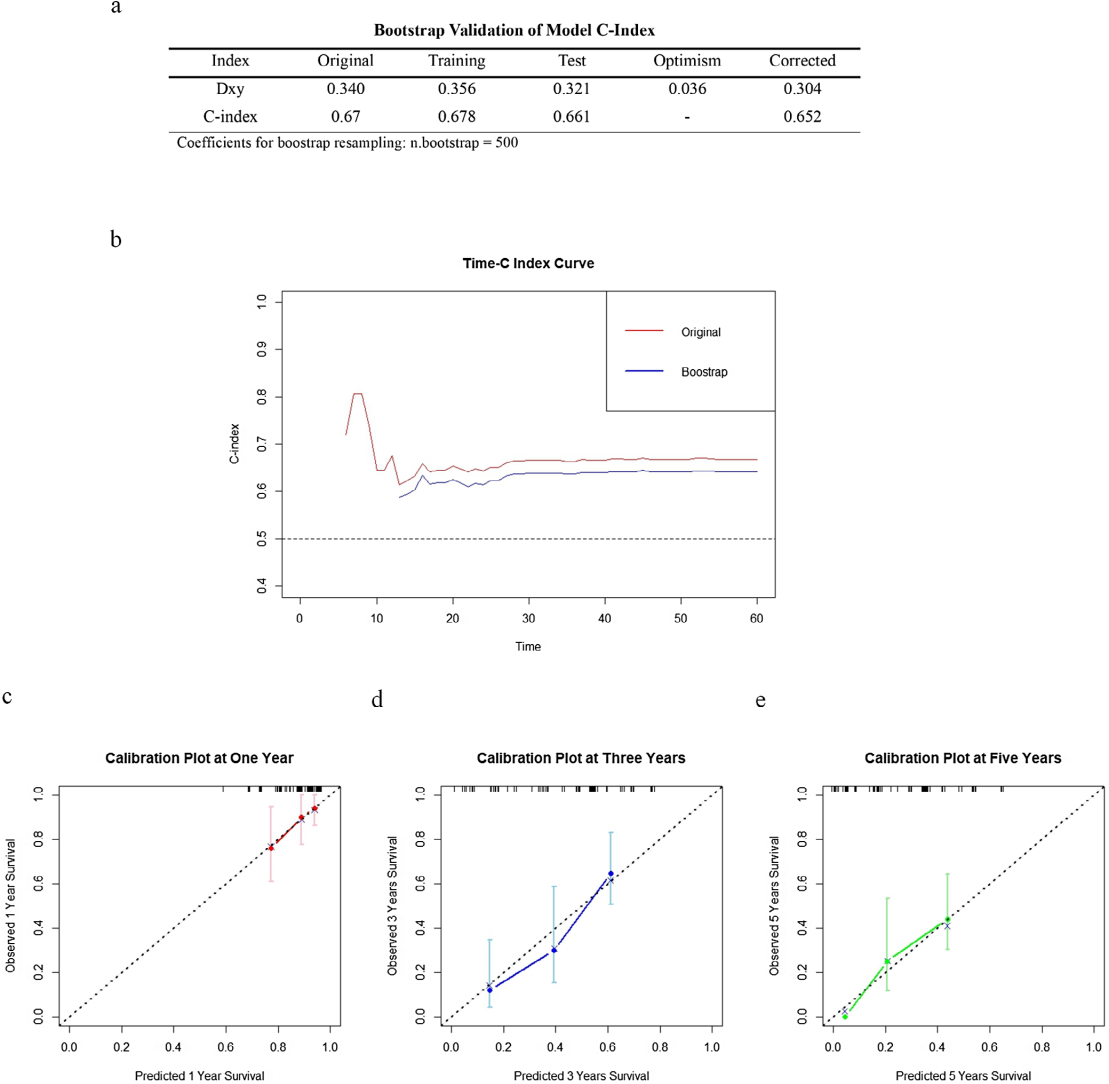
Validation of the prediction model by bootstrap resampling (*n* = 500). (a) Original versus corrected Somers' Delta (Dxy) and concordance index (c‐index) of prediction model were estimated before and after bootstrapping respectively. (b) Time C‐index curves of original versus bootstrapped model showed minimal difference. (c–e) Calibration plot of bootstrapped model showed strong correlation between predicted and observed survival probabilities.

The prediction model was internally validated by bootstrap resampling (*n* = 500) [30]. As shown in Figure 6a, c‐index was calculated based on Somer's delta (Dxy) for the original (Dxy = 0.340, c‐index = 0.67) and the bootstrap corrected models (Dxy = 0.304, c‐index = 0.652), which were similar between the models and suggested a similar degree of discrimination on validation. The change of c‐index over time for both the original and the bootstrapped model was assessed by Time C‐index curve, and they were again similar up to 5 years post‐resection (Figure 6b).

Calibration was also assessed for the bootstrapped model (*n* = 500). The predicted survival probabilities were like the observed survival probabilities at 1, 3, and 5 years (Figure 6c–e). Together, these results suggested that the prediction model performed well with internal validation for discrimination and calibration.

### 3.6 Assessment of the Potential Clinical Benefits of the Prediction Model

**FIGURE 7:**
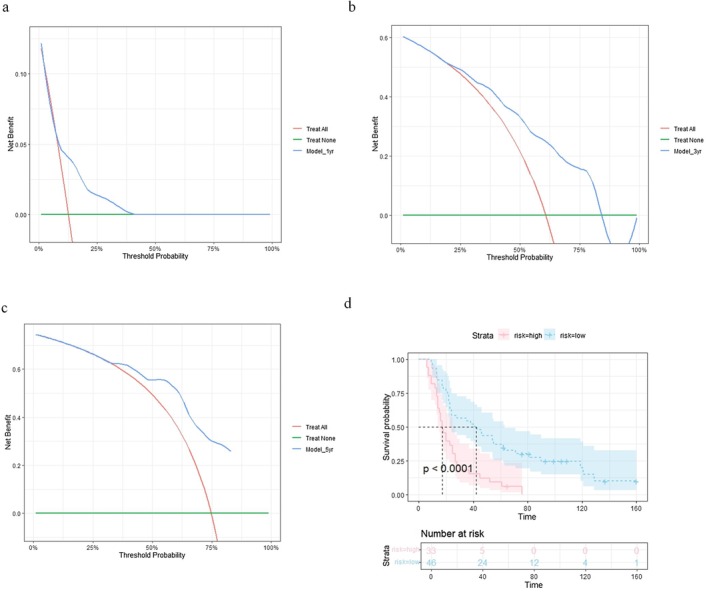
Decision curve analysis and Kaplan Meier curve of the prediction model. (a–c) Decision curve analysis (DCA) at one year, three years and five years showed net benefit of prediction model. (d) Kaplan–Meier (KM) Curve showed statistically significant difference in overall survival between patients with high versus low calculated risk scores.

Decision curve analysis (DCA) is a known method to estimate the potential benefit of a prediction model in clinical practice [29]. Figure 7a–c showed the DCA of the prediction model at 1, 3, and 5‐year post‐resection, respectively. The model outperformed either “treat none” or “treat all” strategies over the range of threshold probabilities at all three time points, suggesting its application may lead to clinical benefit (Figure 7a–c). We further categorised the patients into low versus high risks based on calculated risk scores by the prediction model and compared the two groups by Kaplan–Meier curve (Figure 7d). The high‐risk group had significantly worse overall survival in comparison to the low‐risk group by log‐rank test (*p* < 0.0001) (Figure 7d).

## 4 Discussion

A large proportion of pancreatic cancer patients experience cancer recurrence after surgical resection, leading to poor overall survival even with resectable disease [2]. Therefore, the ability to predict overall survival in patients after surgical resection is important for subsequent therapies. Multiple clinical variables have been shown to act as prognosticators for long‐term survival in PDA [35]. Immunohistochemistry has been used in clinical settings to determine levels of prognostic markers. Previous studies have identified a role of histological prognosticators (such as TUBB3 and hENT1) in predicting post‐resection survival of PDA patients and their response to existing chemotherapy regimens [36]. Cancer immune evasion plays an important role in PDA tumorigenesis, especially by affecting T‐cell functions [3]. In this study, we demonstrated a protocol for integrating clinical data with immune markers identified by mIHC to develop a prediction model of overall survival for patients after resection, which will facilitate further development of a complex prediction model.

Intra‐tumoral CD4+ and CD8+ T cells are independent prognosticators for PDA overall survival [8, 9, 37, 38]. Our results confirmed that a high CD8+ T cell to tumour cell ratio is positively correlated with overall survival, but an opposite effect was demonstrated for CD4+ T cells. This is potentially due to differences in CD4+ T cell differentiation, as regulatory T cell (Treg) infiltration was known to correlate with poor prognosis [37]. Interaction between cancer cell surface MHC I and T cell receptor of CD8+ T cell forms the basis of CD8+ T cell activation, and high MHC I expression was found to correlate with improved patient survival in PDA [31, 39]. We recently reported a role of cancer cell autophagy and PAK4 expression in regulating cell surface MHC I expression and CD8+ T cell infiltration [5, 6]. Here, we built a clinicopathological model connecting patient survival to markers identified from TMA samples, including T cell markers, PAK4, LC3B and MHC I.

Given the important role of immune cells in PDA tumorigenesis, studies have assessed the potential of building prediction models based on PDA TME characteristics [10, 11, 12]. By using single IHC for model development and multiplex IHC for validation, Mahajan et al. [11] developed a histological signature based on immune cell markers and stromal subtypes which predicted progression‐free survival in PDA. This was taken further by two other studies that integrated histological findings with clinical results in a prediction model building [10, 12]. A 2023 study used CODEX spatial protein profiling of 437 PDA patients' TMA cores and integrated it with clinical metadata by six machine learning algorithms for model development [12]. While this approach produced a highly discriminative model (AUC above 0.9), the limited access to the CODEX platform and advanced data science skills may limit its translation into clinical practice [12]. On the other hand, Chen et al. [10] applied multiplex IHC of T cell markers to TMA cores of 80 PDA patients and developed a prediction model based on clinical data and multiplex IHC results using univariate and multivariable Cox PH regression for variable selection. However, the strong potential of confounding effects and multicollinearity between IHC variables subject it to bias for variable selection. As demonstrated in our results, the CD8+ T cell to tumour cell ratio failed to enter the final model with LASSO regression, although it was shown to be an independent prognosticator by multivariable Cox PH regression.

In this study, we streamlined the process for multiplex IHC TMA analysis and developed a pipeline including unmixing, cell phenotyping and spatial analysis using measures that are readily available through commercial software. Conversion of multiplex IHC variables to binary variables was performed due to the semiquantitative nature of the method and a linearity assumption was used in Cox PH regression for continuous variables. The results of multiplex IHC were then combined with clinical variables to enter regularised regression for variable selection to avoid bias. LASSO regression was applied in this study, but other methods such as ridge regression or elastic net regularisation can also be considered depending on the scenario [40, 41]. The prediction model developed can then be assessed for its performance and validated either internally or externally. With a small panel of multiplex IHC markers as well as a limited number of clinical variables, our prediction model was able to discriminate events from non‐events with reasonable accuracy and performed well under internal validation.

Our current prediction model does have limitations. First, the model's ability for discrimination and calibration can be improved by including a larger panel of predictor variables for selection. For multiplex IHC variables, this requires a more comprehensive selection of immune cell annotation markers (e.g., FoxP3, PD1, PDL1, etc.) to distinguish immune cell subsets. Also, important clinical indicators of general health (such as ASA classification and ECOG score) and biomarkers (such as CA19‐9) will need to be included due to their effects on patient survival. Second, the small sample size may lead to selection bias, unreliable estimation of correlation coefficients, and over‐fitting of the model, which requires further validation with a larger population as well as external validation with an independent population before clinical translation becomes practical. Third, although we included adjuvant chemotherapy as a potential predictor variable for model development, we did not conduct a sub‐group analysis based on the chemotherapy regimen and duration of treatment received. This is mainly due to missing information on the details of the chemotherapy regimen and the large variety of regimens used for study participants. With the need for complete case analysis, this will further reduce the sample size and thus power to detect difference. Studies with larger sample sizes will be required to overcome this. Finally, we did not directly compare our model performance with the two existing models due to the lack of a device (such as CODEX platform) and an external validation cohort, which may also be addressed by future research.

While our current model is limited by multiple factors as summarised above, the streamlined protocol for model development served as a backbone for future prediction model development based on mIHC results. A modified and validated clinicopathological prediction model will facilitate risk stratification for patients after resection, thus guiding adjuvant therapy. It may also help to identify subgroups of patients who are responsive to immunotherapy.

In conclusion, we demonstrated a simple and effective protocol for combining mIHC results and clinical data into a prediction model for the overall patient survival after PDA resection. Our results indicated that MHC I intensity, CD4+ T cell to tumour cell ratio, tumour resection margin status, and tumour T stage are the important predictors of overall survival. This prediction model, developed by Cox PH regression, demonstrated a moderate level of discrimination and good calibration in our samples. The protocol provided a framework for developing a future prediction model.

## Author Contributions

**Yi Ma:** conceptualization (equal), formal analysis (lead), funding acquisition (supporting), investigation (lead), methodology (lead), software (lead), validation (equal), writing – original draft (lead), writing – review and editing (lead). **Eunice Lee:** methodology (supporting), software (supporting), validation (supporting), writing – review and editing (supporting). **Khashayar Asadi:** methodology (supporting), resources (equal), writing – review and editing (supporting). **Mehrdad Nikfarjam:** funding acquisition (lead), investigation (equal), supervision (supporting), writing – review and editing (supporting). **Hong He:** conceptualization (equal), funding acquisition (lead), investigation (lead), methodology (equal), supervision (lead), writing – review and editing (lead).

## Funding

This work was supported by grants from Pancare Foundation, Austin Medical Research Foundation (HH‐2023, MN‐2022), and MDHS (Medicine Dental Health Science, University of Melbourne) Seeding Ideas Grants (MN2020, HH, and CA 2021).

## Ethics Statement

The collection of patient's clinical information and generation of human PDA tissue microarray (TMA) were approved by the Austin Health Human Research Ethics Committee (HREC/73948/Austin‐2021).

## Consent

The authors have nothing to report.

## Conflicts of Interest

The authors declare no conflicts of interest.

## Supporting information


**Table S1:** Primary antibodies for multiplex immunohistochemistry.
**Table S2:** Primary antibody and Opal fluorophore pairs for multi‐cycle staining.
**Table S3:** Variance inflation factor for continuous variables.
**Table S4:** Variance inflation factor for binary variables.
**Table S5:** Univariate and Multivariate Cox Regression Results of AJCC Stage.
**Figure S1:** Principal component analysis (PCA) of mIHC variables as continuous variable. (a) D Scree plot demonstrating selection of number of principal components (PC) by elbow method. (b) Correlation of variables to each component by varimax rotation of PCA (i.e., RC). CD8, CD4 and tumour related markers are in blue, yellow, and purple colours respectively. Red arrow represents positive correlation and green arrow represents negative correlation with correlation coefficients included. (c) Scatter plot showing the correlation between each variable to component 1 and component 2.
**Figure S2:** Kaplan Meier (KM) curve showing survival analysis of study participants based on AJCC (edition 8) stage of pancreatic cancer. *p*‐value of log‐rank test was shown on graph with two‐sided *p*‐value < 0.05 considered significant.
**Figure S3:** Assessment of performance of AJCC (edition 8) stage in predicting overall survival. (a–c) Time dependent Receiver Operating Characteristic (ROC) curve of predictive model at one year, three years and five years with Area Under Curve (AUC) as calculated. (d) One year, three years and five years ROC curve was combined for direct comparison.

## Data Availability

Data and study material will be available upon request from the corresponding author.
