# Supplementary material for: A Streamlined Protocol for Developing a Clinicopathological Prediction Model for Patient Survival of Post‐Resection of Pancreatic Cancer
Source: Cancer Med. 2026 Jan 28;15(2):e71535. doi: 10.1002/cam4.71535 (PMC12848901; doi:10.1002/cam4.71535)
Supplement: Supplementary file 1 — Table S1: Primary antibodies for multiplex immunohistochemistry. Table S2: Primary antibody and Opal fluorophore pairs for multi‐cycle staining. Table S3: Variance inflation factor for continuous variables. Table S4: Variance inflation factor for binary variables. Table S5: Univariate and Multivariate Cox Regression Results of AJCC Stage. Figure S1: Principal component analysis (PCA) of mIHC variables as continuous variable. (a) D Scree plot demonstrating selection of number of principal components (PC) by elbow method. (b) Correlation of variables to each component by varimax rotation of PCA (i.e., RC). CD8, CD4 and tumour related markers are in blue, yellow, and purple colours respectively. Red arrow represents positive correlation and green arrow represents negative correlation with correlation coefficients included. (c) Scatter plot showing the correlation between each variable to component 1 and component 2. Figure S2: Kaplan Meier (KM) curve showing survival analysis of study participants based on AJCC (edition 8) stage of pancreatic cancer. p‐value of log‐rank test was shown on graph with two‐sided p‐value < 0.05 considered significant. Figure S3: Assessment of performance of AJCC (edition 8) stage in predicting overall survival. (a–c) Time dependent Receiver Operating Characteristic (ROC) curve of predictive model at one year, three years and five years with Area Under Curve (AUC) as calculated. (d) One year, three years and five years ROC curve was combined for direct comparison. [file CAM4-15-e71535-s001.docx]

| **Table S1. Primary antibodies for multiplex immunohistochemistry** | | | |
| --- | --- | --- | --- |
| Protein target | Dilution | Cat. number | Company |
| PAK4 | 1:100 | PA5-15120 | Invitrogen |
| CK19 | 1:2000 | ab52652 | Abcam |
| LC3B | 1:1000 | 3868 | Cell Signaling & Technology |
| CD4 | 1:500 | ab288724 | Abcam |
| CD8 | 1:2000 | ab237709 | Abcam |
| MHC I | 1:1000 | ab134189 | Abcam |

| **Table S2. Primary antibody and Opal fluorophore pairs for multi-cycle staining** | | | |
| --- | --- | --- | --- |
| Cycle | Primary antibody | Opal fluorophore | Dilution of fluorophore |
| 1 | PAK4 | Opal 520 | 1:100 |
| 2 | CK19 | Opal 690 | 1:200 |
| 3 | LC3B | Opal 570 | 1:200 |
| 4 | CD4 | Opal 620 | 1:100 |
| 5 | CD8 | Opal 540 | 1:100 |
| 6 | MHC I | Opal 650 | 1:200 |

| **Table S3. Variance inflation factor for continuous variables** | |
| --- | --- |
| Variable | VIF |
| Age | 1.889 |
| Sex   - Male - Female | -  1.909 |
| PAK4 Intensity | 4.402 |
| LC3B Intensity | 3.798 |
| MHC I Intensity | 3.009 |
| CD4: CK19 Ratio | 9.093 |
| CD8: CK19 Ratio | 12.039 |
| CD4:CK19 Pair-Wise Distance | 8.607 |
| CD8:CK19 Pair-Wise Distance | 9.109 |
| CD4:CK19 Minimum Distance | 5.158 |
| CD8:CK19 Minimum Distance | 3.066 |
| %CD4 within 50µM from CK19 | 9.895 |
| %CD8 within 50µM from CK19 | 8.867 |
| CD4:CK19 Normalised Mixing Score | 13.177 |
| CD8:CK19 Normalised Mixing Score | 12.755 |
| Cancer Site   - Head/Neck - Body/Tail - Multifocal | -  1.629  3.379 |
| Resection Margin   - R0 - R1 | -  2.021 |
| T Stage | 1.690 |
| N Stage | 1.975 |
| Grade | 2.166 |
| Lymphovascular Invasion   - No - Yes | -  1.861 |
| Perineural Invasion   - No - Yes | -  1.938 |
| Portal Vein Resection   - No - Yes | -  2.205 |
| Adjuvant Chemotherapy   - No - Yes | -  1.905 |
| Abbreviations: VIF: Variance inflation factor. | |

| **Table S4. Variance inflation factor for binary variables** | |
| --- | --- |
| Variable | VIF |
| Age   - Below 65 - Above 65 | -  1.353 |
| Sex   - Male - Female | -  2.043 |
| PAK4 Intensity   - Low - High | -  2.316 |
| LC3B Intensity   - Low - High | -  2.777 |
| MHC I Intensity   - Low - High | -  2.314 |
| CD4: CK19 Ratio   - Low - High | -  2.656 |
| CD8: CK19 Ratio   - Low - High | -  4.702 |
| CD4:CK19 Pair-Wise Distance   - Low - High | -  3.297 |
| CD8:CK19 Pair-Wise Distance   - Low - High | -  2.875 |
| CD4:CK19 Minimum Distance   - Low - High | -  3.290 |
| CD8:CK19 Minimum Distance   - Low - High | -  3.373 |
| %CD4 within 50µM from CK19   - Low - High | -  4.455 |
| %CD8 within 50µM from CK19   - Low - High | -  4.545 |
| CD4:CK19 Normalised Mixing Score   - Low - High | -  4.553 |
| CD8:CK19 Normalised Mixing Score   - Low - High | -  4.780 |
| Cancer Site   - Head/Neck - Body/Tail - Multifocal | -  2.194  1.857 |
| Resection Margin   - R0 - R1 | -  1.931 |
| T Stage | 2.207 |
| N Stage | 2.098 |
| Grade | 2.042 |
| Lymphovascular Invasion   - No - Yes | -  1.721 |
| Perineural Invasion   - No - Yes | -  2.155 |
| Portal Vein Resection   - No - Yes | -  2.127 |
| Adjuvant Chemotherapy   - No - Yes | -  1.807 |
| Abbreviations: VIF: Variance inflation factor. | |

| **Table S5. Univariate and Multivariate Cox Regression Results of AJCC Stage** | | | | | | |
| --- | --- | --- | --- | --- | --- | --- |
|  | Univariate | | | Multivariate | | |
| Variable (n=79) | Hazard ratio | 95% CI | p-value | Hazard ratio | 95% CI | p-value |
| Stage   - 1A - 1B - 2A - 2B - 3A - 3B - 4 | -  1.61  2.01  2.26  8.57  2.33  - | -  0.53-4.87  0.69-5.86  0.76-6.66  1.78-41.2  0.26-21.3  - | -  0.4  0.2  0.14  0.007  0.5  - | -  4.34  2.38  3.22  3.03  6.01  - | -  1.06-17.8  0.53-10.6  0.71-14.5  0.46-20.0  0.26-140  - | -  0.042  0.3  0.13  0.2  0.3  - |
| Abbreviations: CI: Confidence Interval. Multivariate analysis includes all study variables. | | | | | | |

**Supplementary figure legend**


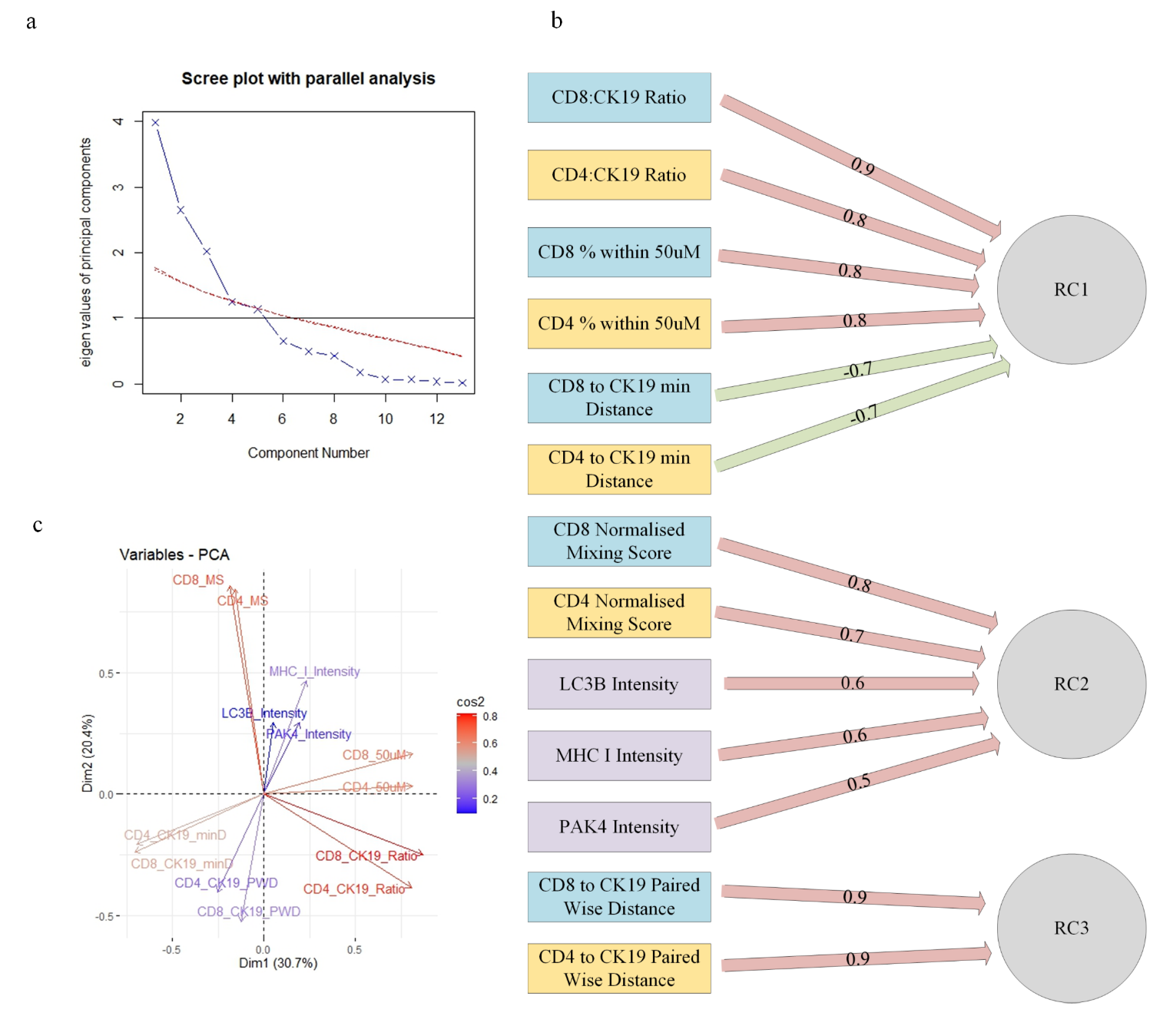


**Figure S1.** Principal component analysis (PCA) of mIHC variables as continuous variable. **a)** D Scree plot demonstrating selection of number of principal components (PC) by elbow method. **b)** Correlation of variables to each component by varimax rotation of PCA (i.e. RC). CD8, CD4 and tumour related markers are in blue, yellow, and purple colours respectively. Red arrow represents positive correlation and green arrow represents negative correlation with correlation coefficients included. **c)** Scatter plot showing the correlation between each variable to component 1 and component 2.


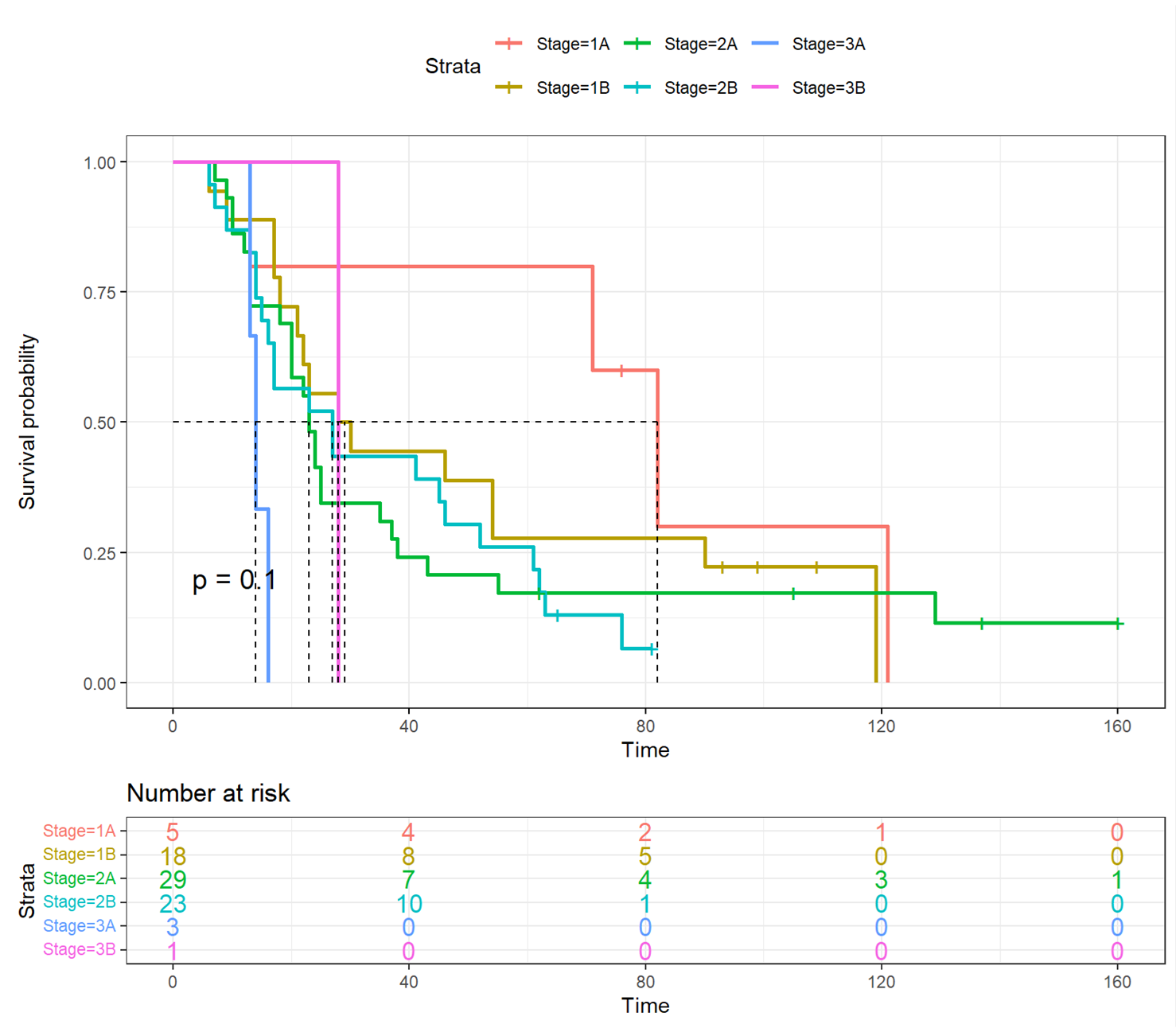


**Figure S2.** Kaplan Meier (KM) curve showing survival analysis of study participants based on AJCC (edition 8) stage of pancreatic cancer. P-value of log-rank test was shown on graph with two-sided p-value <0.05 considered significant.


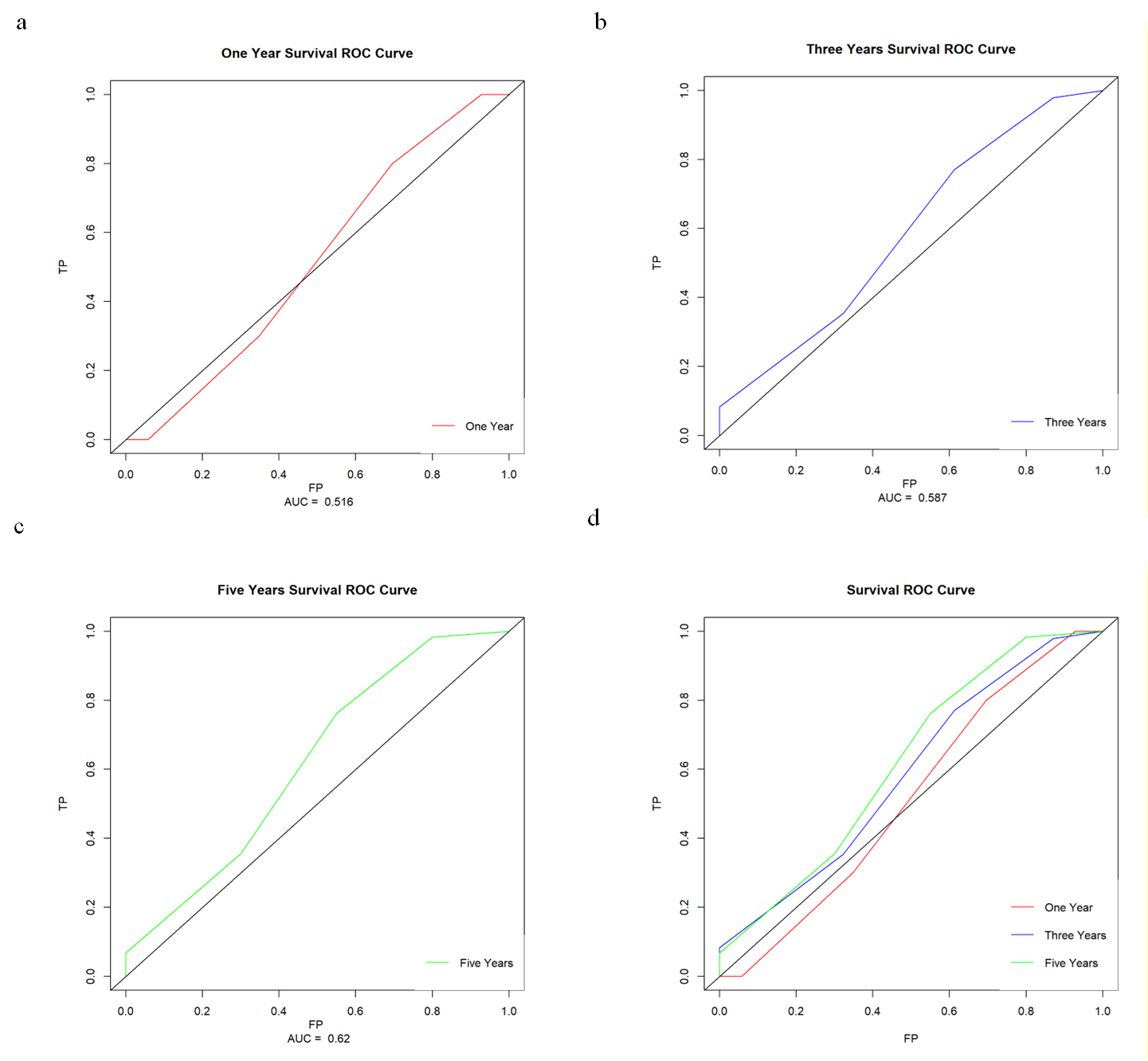


**Figure S3.** Assessment of performance of AJCC (edition 8) stage in predicting overall survival. **a-c)** Time dependent Receiver Operating Characteristic (ROC) curve of predictive model at one year, three years and five years with Area Under Curve (AUC) as calculated. **d)** One year, three years and five years ROC curve was combined for direct comparison.
